# Novel imaging approach for simultaneous tracking of cell dynamics in distinct tissue layers reveals cells involved in colonic peristalsis

**DOI:** 10.3389/fimag.2025.1538533

**Published:** 2025-04-02

**Authors:** Salah A. Baker, Peter J. Blair, Sharif Amit Kamran, Kenton M. Sanders

**Affiliations:** Department of Physiology and Cell Biology, University of Nevada School of Medicine, Reno, NV, United States

**Keywords:** deep tissue imaging, confocal microscopy, calcium imaging, dynamic fluorescence signals, simultaneous tissue layer imaging

## Abstract

We have developed a novel approach for high-resolution confocal imaging across multiple tissue planes simultaneously. By combining confocal microscopy, piezo actuators, and optogenetic sensors, we can simultaneously capture images of dynamic fluorescence signals from various cell populations in different tissue layers (Z planes). This enables the decoding of cell-to-cell communication through complex tissues, offering a significant advancement in understanding how cells in distinct layers of tissue communicate and coordinate their functions and produce integrated behaviors. For example, our technique sheds light on myogenic coordination underlying colonic motility. Examining various cell types, such as interstitial cells of Cajal (ICC) and smooth muscle cells (SMC), distributed through the thickness of muscle layers, we demonstrate distinct Ca^2+^ signaling patterns and organization that underlie complex colonic motor activities.

## Graphical Abstract

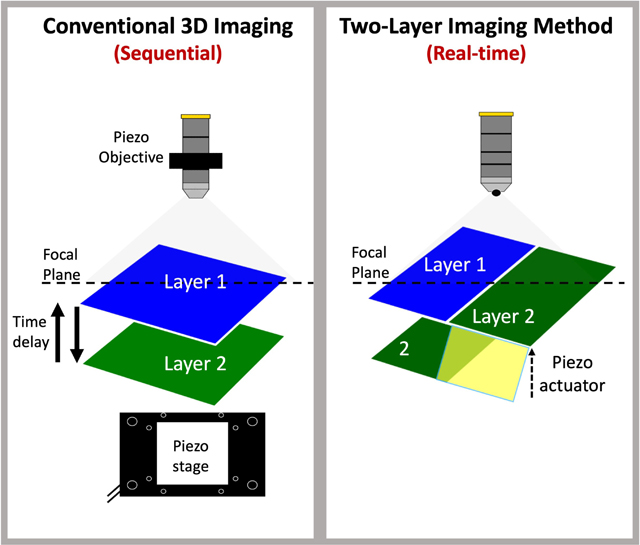

Novel simultaneous two-layer imaging: Conventional confocal imaging of multi-layered tissues relies on sequential acquisition (left panel), requiring either movement of the sample or objective along the z-dimension to capture a single focal plane at a time. This process introduces a time delay when switching between layers, affecting temporal resolution. In contrast, the new two-layer imaging method achieves simultaneous imaging of two layers without delay by utilizing a piezo actuator, enabling the effective capture of both layers at the same focal plane in real time.

## 1. Introduction

Live cell fluorescence imaging is a widely used tool in medical and research applications and provides mechanistic information on cellular functions and regulation. Dynamic cellular imaging requires speed and sensitivity, and if effective can provide readouts of various cellular responses, including ion fluxes, pH, protein trafficking, Ca^2+^ or voltage. Subcellular signals are complex in nature. For example, cellular voltage and Ca^2+^ signaling patterns differ depending on cell type, cellular compartmentalization, and target tissue, but these signals are interrelated (Roome and Kuhn, 2018; Berridge et al., 2003). Thus, the ability to evaluate these signals simultaneously with high spatial and temporal resolution is an important advancement for which to strive. To understand how cellular signaling generates tissue- or organ-wide integrated behaviors, imaging of dynamic signals within complex tissues requires precise cellular localization of signal. This imaging requires adequate signal intensity (e.g., signal-to-noise resolution), spatial and temporal definition and the molecular entities that create and regulate the signals but also their temporal characteristics, which are vital to achieving a desired cellular response.

Several platforms are available for dynamic cellular imaging of signals within tissues, such as confocal microscopy which uses laser scanning and pinholes to improve the focus and resolution of images by blocking out-of-focus light. Confocal imaging has higher resolution than traditional widefield microscopy and allows for microsecond acquisition with low phototoxicity and photobleaching. Confocal microscopy is a powerful imaging technique that enables acquisition of static or dynamic fluorescence signals at different depths of tissue due to its inherent property of optical sectioning. It can optically section thick samples with high resolution into thinner sections of <1 μm (Jonkman et al., 2020), facilitating the visualization of sample sections in the Z dimension at a given time in a sequential manner, and can provide a 3D reconstitution by acquiring multiple images in the Z dimension. This enables researchers to identify the quantitative and spatial distribution of molecules within a given tissue.

One major obstacle for fluorescence imaging methods is that tissues are often organized in layers, with each layer containing specialized cell populations with different functions. Layering of specific types of cells is critical for the proper functioning of complex tissues, and confocal microscopy is a powerful tool that can optically section multilayer samples with high resolution. For dynamic fluorescence signal acquisition, such as Ca^2+^ imaging, confocal microscopy can provide information on a single plane of the multilayer tissue over a given time, as the field of view (FOV) is visualized in x-y dimensions. This allows accumulation of information about cells in a single tissue layer within the thickness of the tissue. Traditional confocal imaging methods fall short when applied to dynamic processes like real-time Ca^2+^ imaging. These methods rely on sequential image acquisition that introduces time delays which prevent the capture of rapid cellular interactions occurring across different depths within tissue layers. This limitation hampers the ability to decode cell-to-cell communication between different tissue layers. Planar confocal microscopy, while useful for certain applications, is not capable of providing real-time, simultaneous imaging across multiple layers. Consequently, it offers only limited insights into how cells in distinct functional layers communicate and coordinate their activities to drive integrated tissue responses.

This limitation has led us to develop a new method that can image cells in separate tissue layers in real-time and bringing two distinct layers into the same focal plane using cost effective and commercially available piezo actuators that can be implemented with new or existing confocal microscopes. The proposed method utilizes a combination of confocal imaging, piezo actuators and fluorescent indicators of cell signaling (i.e., optogenetic sensors or fluorescent tags) that are used widely in the field of cell biology. This approach allows visualization of cells in different tissue layers simultaneously and overcomes some of the limitations of standard confocal microscopy that rely on sequential imaging acquisition.

## 2. Materials and equipment

### 2.1 Ethics statement

The animals used, protocols performed and procedures in this study were in accordance with the National Institutes of Health Guide for the Care and Use of Laboratory Animals and approved by the Institutional Animal Use and Care Committee at the University of Nevada, Reno (IACUC; Protocol: 1131–1).

### 2.2 Animals

GCaMP6f-floxed mice [Ai95 (RCL-GCaMP6f)-D], Myh11-Cre-eGFP (Xin et al., 2002) and C57BL/6 mice, their wild-type siblings, were purchased from Jackson Laboratories (Bar Harbor, MN, USA). Kit-iCre mice (c-Kit^+*/Cre-ERT2*^) were gifted from Dr. Dieter Saur (Technical University Munich, Munich, Germany), Kit^+/*copGFP*^ mice were bred in house.

#### 2.2.1 Kit-iCre-GCaMP6f/Acta2-RCaMP1.07 mice

*Acta2-RCaMP1.07* mice [*tg(RP23–370F21-RCaMP1.07)B3–3Mik/J*] express the fluorescent Ca^2+^ indicator RCaMP1.07 in SMCs under the control of the Acta2 locus promoter/enhancer regions. These animals were obtained from Jackson Laboratories (Bar Harbor, MN, USA). To generate cell-specific expression in two distinct cell types (ICC and SMCs) *Acta2-RCaMP1.07* mice were bred with *Kit*^*Cre*−*ERT*2^*/GCaMP6f*^*fl*/*fl*^ mice. The offspring *Kit-iCre-GCaMP6f/Acta2-RCaMP1.07* mice were identified by genotyping after receiving tamoxifen which served to delete the STOP cassette in the Cre-expressing cells; resulting in the expression of the fluorescent Ca^2+^ indicator protein, GCaMP6f. These mice allowed simultaneous, dual color imaging of ICC and SMCs. iCre mice were injected with tamoxifen (TAM; Intraperitoneal injection; IP) at 6–8 weeks of age (2 mg of TAM for three consecutive days), as described previously (Baker et al., 2021).

### 2.3 Tissue preparation

After the mice were anesthetized by isoflurane (AErrane; Baxter, Deerfield, IL, USA) and killed by cervical dislocation, an abdominal incision was made and the GI organs removed and placed in Krebs-Ringer bicarbonate (KRB) solution. Short segments (2 cm) were taken from the small intestine and proximal colon, and cut open along the mesenteric border. Any intraluminal contents were washed away with KRB. Using fine forceps, the mucosa was carefully peeled away to leave the intact *tunica muscularis*, which was then used in the following experiments.

### 2.4 Immunohistochemistry

Intact muscle sheets from wild-type small intestines were pinned to the Sylgard base of a fixing dish. KRB was removed and replaced with 4% paraformaldehyde (30 min, room temp). Tissues were then unpinned and placed in a test tube containing phosphate-buffered saline with 0.1% Tween 20 (PBS-T). The tissues were washed on a rotator for at least 5 h, changing the PBS-T every hour. After washing, the tissues were placed in a blocking solution (10% normal donkey serum in 0.5% Triton-X), to minimize non-specific antibody binding. After block, tissues were directly transferred to a solution of primary antibody and incubated for 48 h at 4°C. The primary antibody used in this study was a polyclonal raised against c-Kit (mSCFR, AF1356, R and D Systems, MN, USA; 1:100 dilution in 0.5% Triton-X working solution). Next, the tissues were washed in PBS-T for at least 5 h before being incubated in a secondary antibody, Alexa Fluor 488-conjugated anti-goat IgG (1:1000 in PBS-T; Jackson ImmunoResearch, #705–545-147, PA, USA), for 1 h at room temperature. Tissues were again washed in PBS-T for at least several hours before being mounted on glass slides with Vectashield Vibrance Mounting Medium (H-1800, Vector Laboratories, Inc., CA, USA). A Nikon A1R confocal microscope (Nikon Instruments Inc., NY, USA) was used to obtain Z-stack images of ICC and Z-stack projections were then produced using Image J software (National Institutes of Health, MD, USA, http://rsbweb.nih.gov/ij). The distance between ICC subtypes was calculated at multiple regions on several tissue preparations. The total number of regions is referred to as *n* throughout the manuscript.

### 2.5 Piezo electric actuator

A piezo unimorph bending actuator (Python^™^ PUA3008–5H200) from Bimitech Inc. (San Jose, CA, USA) was used. The piezo measured 46 × 8 × 0.54 mm, and is capable of producing a displacement of up to 0.42 mm. We also had a custom piezo created that measured 19 × 8 × 0.54 mm. Python^™^ actuators are comprised of a piezo layer that is hermetically sandwiched between two durable epoxy resin layers, which is then further coated with two thin silicone rubber layers. This structure makes the piezo water resistant and very durable. The Python^™^ piezo actuator was operated by a Thor labs (Newton, NJ, USA) piezo controller (MDT693). The MDT693 is a precision, low-noise, low-drift, high voltage controller for piezo actuators. It has three channels that allow it to control multiple devices or multiple axis piezo stages. However, to control our single Python^™^ piezo bender, only one channel was required. The front panel of the MDT693 features precision 10-turn potentiometers for the precise manual setting of the outputs over the full operating range (0–150V). The device is utilized to produce 2 Z plane imaging in combination with spinning disk confocal microscopy.

### 2.6 Ca^2+^ imaging

The bare ends of the wires in a BNC cable were soldered to the appropriate terminals on the piezo actuator. The bare wire connections were then waterproofed by sealing with silicone caulk (Henkel, CT, USA). Once the caulk was fully cured (after 24 h), the piezo was attached to the bottom of a Sylgard-coated dish with marine-grade waterproof epoxy (J-B Weld, GA, USA). Furthermore, a thin layer of Sylgard (Dow Chemical Company, MI, USA) 35 mm in diameter was used as a platform around the active end of the piezo, to which we could pin tissues and stretch them across the end of the piezo, while keeping the tissues flat. For experiments, small intestine or colon muscle sheets were pinned so that half of their width was stretched across the piezo and then perfused with KRB solution at 37°C. This was achieved using a perfusion peristaltic pump (minipuls 3, Gilson, WI, USA) with an electric in-line heater and controller (SH-27B and TC-344C, Warner instruments, CT, USA). The piezo BNC cable was attached to the output of a piezo controller and turning the corresponding knob varied the applied voltage. This facilitated precise control of the magnitude of piezo bending. Ca^2+^ imaging was performed using a spinning-disk confocal system (CSU-W1; spinning disk, Yokogawa Electric, Tokyo, Japan) mounted on an upright Nikon Eclipse FN1 microscope equipped with several water immersion Nikon CFI Fluor lenses (10× 0.3 NA, 20× 0.5 NA, 40× 0.8 NA, 60× 0.8 NA and 100× 1.1 NA; Nikon Instruments, New York, USA). The system is equipped with two solid-state laser lines of 488 nm and 561 nm. The laser lines are combined with a borealis system (ANDOR Technology, Belfast, UK) to increase laser intensity and uniformity throughout the imaging FOV. The system also has two high-speed electron multiplying charged coupled devices (EMCCD) cameras (Andor iXon-Ultra 897 EMCCD Cameras; ANDOR Technology, Belfast, UK) to allow dual-color imaging simultaneously and maintain sensitive and fast speed acquisition at full frame of 512 × 512 active pixels as previously described (Baker et al., 2021). Briefly, image sequences were collected at 33 to 50 fps using MetaMorph software (MetaMorph Inc, TN, USA).

### 2.7 Ca^2+^ imaging analysis

Movies of two layers (2-Z planes) Ca^2+^ transients in ICC and SMCs were imported, preprocessed and analyzed using Fiji/Image J (National Institutes of Health, MD, USA, http://rsbweb.nih.gov/ij); Briefly, movies of Ca^2+^ transients (stacks of TIFF images) were imported into Fiji and background subtracted and smoothed (Gaussian filter: 1.5 × 1.5 μm, StdDev 1.0). The Ca^2+^ signals were plotted in 2D Ca^2+^ occurrence maps (x axis= time, y axis= cell space) that give information on their spatial and temporal activation. Signal segmentation and analysis were preformed using an automated machine learning plugin (STMapAuto: https://github.com/gdelvalle99/STMapAuto) and 4SM software (https://github.com/SharifAmit/CalciumGAN/tree/main) as described previously (Leigh et al., 2020; Kamran et al., 2022; Moghnieh et al., 2022).

All means ± standard errors were calculated, and graphs generated, using GraphPad Prism 8.0.1 (San Diego, CA, USA) All figures were created using Powerpoint (Microsoft, WA, USA).

## 3 Methods

### 3.1 Tissue organization

**FIGURE 1:**
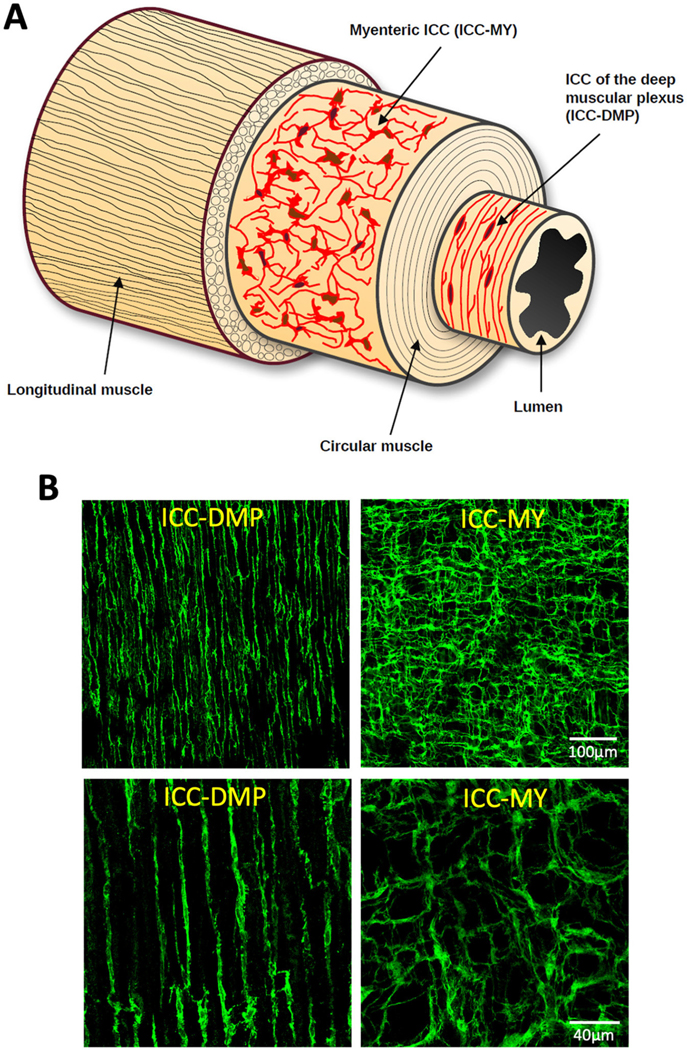
Interstitial cells of Cajal (ICC) distribution across layers of the small intestine. **(A)** Cartoon of the structure of the small intestine showing ICC at the level of the myenteric plexus (ICC-MY) and deep muscular plexus (ICC-DMP) across the small intestine layers. **(B)** Confocal images of murine small intestine that has been labeled with an antibody against mouse stem cell factor receptor (mSCFR; Kit). The images on the left show ICC-DMP, while the ones on the right show ICC-MY. Top images are 20x and the bottom pair are 60x magnification.

Many organs, including those in the gastrointestinal system, are composed of spatially organized layers of cells, which makes it challenging to image and visualize dynamic signals across these layers simultaneously. For example, gastrointestinal tissues have distinct layers, each with specialized cell types that perform different functions, complicating efforts to capture real-time interactions between these layers. Gastrointestinal organs are composed of multiple layers: mucosa for absorption and secretion, submucosa for structural support, interstitial cells and nerve bundles for organization of functions, and circular (CM) and longitudinal (LM) muscle layers for motility. Of interest to us are the interstitial cells of Cajal (ICC) that are arranged in anatomically distinct layers within the myenteric region and circular muscle (Komuro, 2006). In the small intestine, there are two primary types of ICC. One type surrounds the myenteric plexus (ICC-MY), while the other is located within the circular muscle at the deep muscular plexus (ICC-DMP; Figure 1). These distinct subtypes provide different inputs and modes of regulation that facilitate different motor functions in the gut (Sanders et al., 2016, 2024; Drumm et al., 2022; Sanders et al., 2023). In the colon, ICC are more complex as there are four subtypes and each is located at different layers within the *tunica muscularis*: submucosal ICC (ICC-SM) located at the submucosal border, ICC-IM located within bundles of circular muscle, ICC-MY in the myenteric region located within the myenteric plexus and ICC-SS located at the serosal surface.

We developed a technique to image dynamic fluorescence signals in multiple classes of ICC and smooth muscle cells (SMCs) cells in different layers (two Z depths) simultaneously.

### 3.2 Imaging system components

**FIGURE 2:**
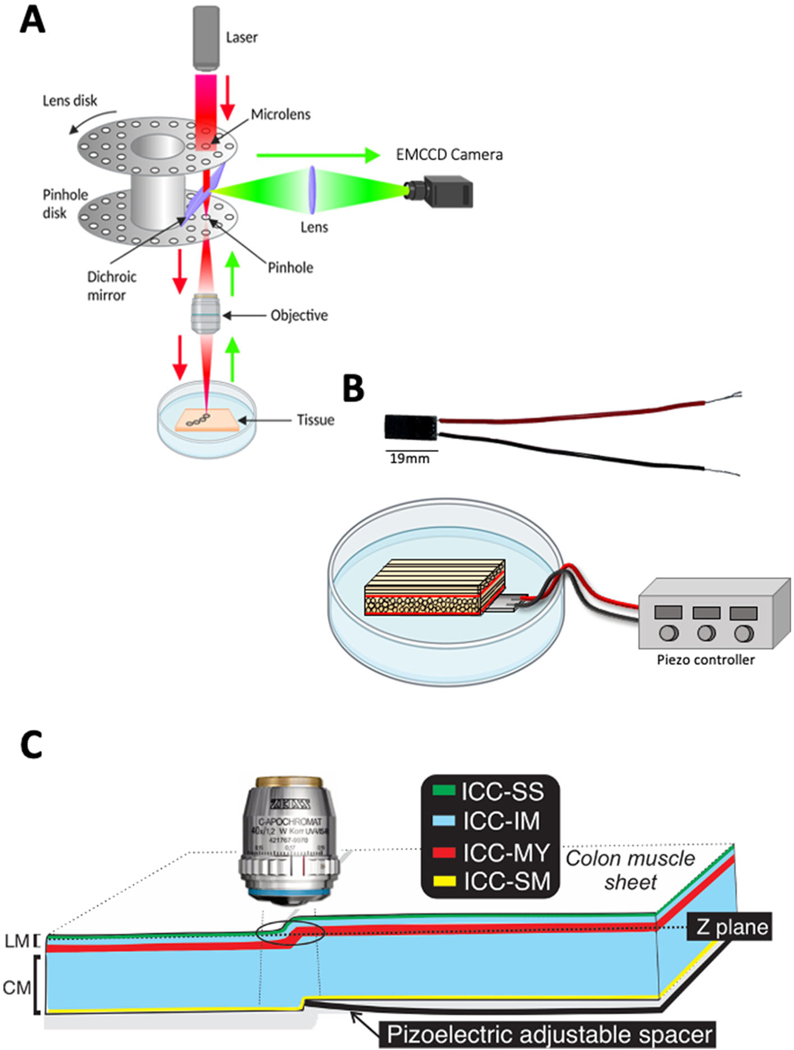
System imaging components. **(A)** Schematic illustrating the light path of a spinning disk confocal microscope. (B) Photograph of piezo actuator with attached wires **(top)**. Diagram of piezo actuator placed under a sample of small intestine in a petri dish. The piezo wires are attached to a piezo controller **(below)**. **(C)** Diagram showing how the piezo actuator can raise part of the muscle sheet, thus allowing Interstitial cells of Cajal (ICC) in different z-planes to be visualized at the same time. The black ring indicates the field of view (FOV). LM refers to longitudinal muscle and CM refers to the circular muscle. ICC types distribution across colonic muscles are color coded, ICC-SS (subserosal ICC; green), ICC-IM (Intramuscular ICC; blue), ICC-MY (Myenteric ICC; red) and ICC-SM (submucosal ICC; yellow).

Our approach uses a combination of spinning-disk confocal microscopy (Figure 2A) and a piezo actuator (Figure 2B) to image two layers within the bulk of the tissue (2 Z planes) in the same FOV in real-time. The actuator is driven by a piezo controller, to precisely control the degree of displacement by varying the voltage across the piezo stack (Figure 2B, see materials section for details).

The piezo actuator was placed in a Sylgard-coated dish with GI muscles secured as flat sheets above the surface of the piezo actuator (Figure 2B; see materials section for details).

By increasing the voltage across the piezo actuator using the piezo-electric controller, it was caused to move and lift part of the tissue into a different Z plane (Figure 2C). By imaging the area of tissue just above the end of the piezo we could visualize cells in different Z planes, in the same FOV (Figure 2C).

## 4 Results

### 4.1 Approach validation: using non-dynamic fluorescence signals

**FIGURE 3:**
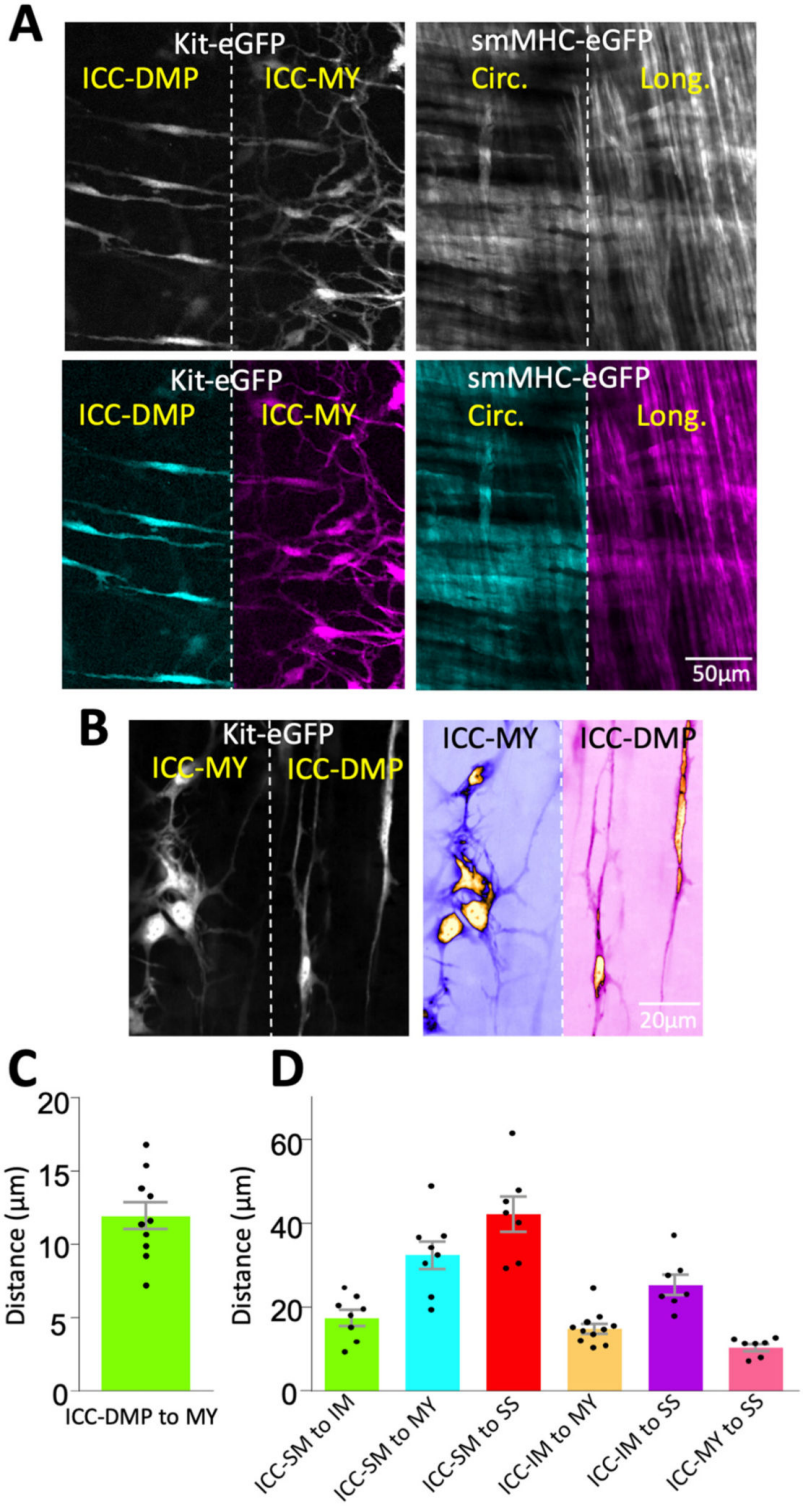
Imaging of ICC and SMCs at different z planes in the same FOV. **(A)** Spinning disk confocal image of Kit-eGFP small intestine after bending with the piezo actuator **(left)**. Pseudocolored versions of the same image to emphasize that both interstitial cells of Cajal in deep muscular plexus (ICC-DMP) and ICC-MY of the myenteric plexus are visible in the same field of view (bottom two images in **A**). Right panel is a pseudocolored image from a smMHC-eGFP small intestine, showing the simultaneous visualization of circular and longitudinal smooth muscle cells (SMCs). **(B)** Images of ICC-DMP and ICC-MY of Kit-eGFP small intestine at high magnification (60x), raw images **(left)** and color coded **(right)**. Note the distinct morphology of each cell type in the same FOV. **(C)** Graph showing the distance between ICC-DMP and ICC-MY in the small intestine. **(D)** Graph showing the distance between various subpopulations of ICC distributed across colonic muscles.

To validate our approach, we first tested the principles of our method on non-dynamic fluorescent signals. Using our method, we achieved two Z plane imaging of eGFP-positive ICC and eGFP-positive SMCs in small intestine muscles distributed in two distinct tissue layers across the depth of the tissue (Figure 3). We simultaneously visualized ICC-DMP and ICC-MY that are located in different layers of muscle within the same FOV (i.e., in different Z planes; Figure 3A, left panels) and imaged SMCs in circular and longitudinal muscle layers (also located in different Z planes; Figure 3A, right panels). Higher magnification images of ICC-DMP and ICC-MY of ICC in the small intestine show the district morphology of each cell type correlating to each tissue layer (Figure 3B). To estimate how much the tissue was displaced by the piezo actuator, the distance between ICC-MY and ICC-DMP was calculated. To achieve this, we used confocal microscopy to visualize each subpopulation of ICC, noted their location in the Z axis, and then calculated the distance between them. In the small intestine, we found that the average distance between ICC-DMP and ICC-MY was 11.9 ± 0.9 μm (Figure 3C, *n* = 10). In the colon, distances between the four types of ICC in the colon were summarized in Figure 3D and Table 1.

### 4.2 Approach validation: using dynamic fluorescence signals

**FIGURE 4:**
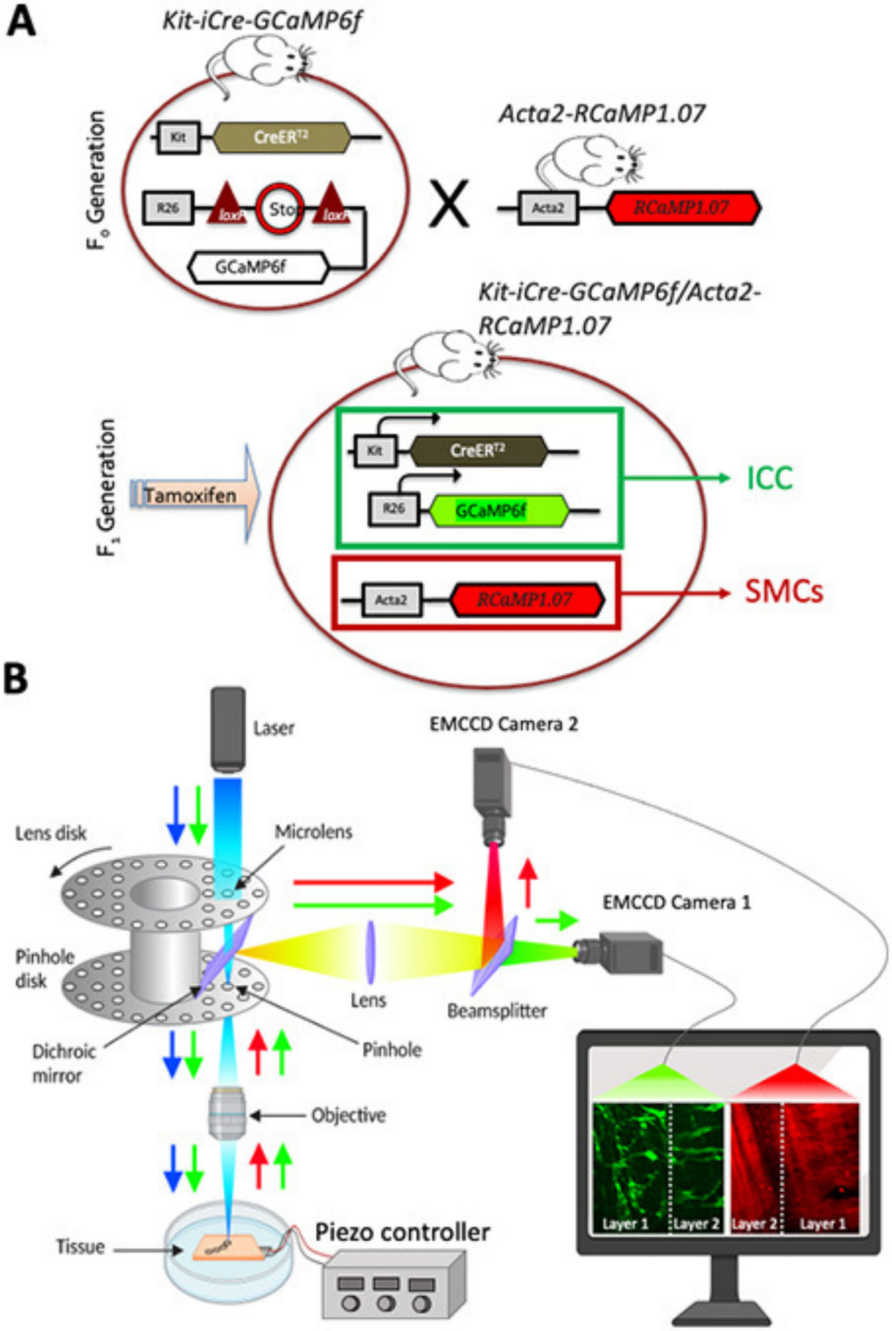
Optogenetic mouse model and dual-color system configuration. **(A)** Schematic diagram showing the breeding to generate cell-specific expression in two distinct cell types (ICC, interstitial cells of Cajal and SMCs, smooth muscle cells). SMCs: *Acta2-RCaMP1.07* mice were bred with ICC: *Kit*^*Cre−ERT2*^*/GCaMP6f*^*fl/fl*^ mice. The offspring (*Kit-iCre-GCaMP6f/Acta2-RCaMP1.07* mice) were then administered tamoxifen to delete the STOP cassette in Cre-expressing cells, resulting in the expression of the fluorescent Ca^2+^ indicator protein GCaMP6f (green) in ICC and RCaMP (red) is expressed in SMCs. These mice allowed simultaneous, dual color imaging of ICC and SMCs. **(B)** Schematic diagram showing image acquisition using spinning-disk confocal system. Dual excitation beams (488nm, blue arrow and 560 nm, green arrow) were used to excite colonic segments that express GCaMPf6f in ICC and RCaMP1.7 in SMCs. Sample fluorescence emissions (green and red) were collected and visualized on two cameras simultaneously. Camera 1 records the green channel from both layers, separated by the white dashed line, while Camera 2 records the red channel from the same two layers, also divided by the white dashed line.

To further validate our technique, we measured dynamic fluorescent signals, such as Ca^2+^, across two distinct tissue layers simultaneously. High-resolution, real-time Ca^2+^ imaging was conducted in multiple classes of interstitial cells of Cajal (ICC) and multiple classes of smooth muscle cells (SMCs) located in different Z planes and organized into two tissue layers. This was achieved using mice genetically engineered to express optogenetic sensors in both ICC (GCaMP6f, a green Ca^2+^ sensor) and smooth muscle cells (SMCs, RCaMP1.07, a red Ca^2+^ sensor; Figure 4A). This dual-sensor system allowed for precise monitoring of Ca^2+^ dynamics in different cellular populations across spatially separated tissue layers. Using a dual color spinning-disk confocal system (Figure 4B; see material section) we were able to resolve dynamics of intercellular Ca^2+^ in multiple cell populations. For example, ICC subtypes (ICC-MY, ICC-IM) express the green GCaMP signal and SMCs subtypes (circular and longitudinal muscle) express the red RCaMP signal. We were able to visualize all these four cell types that are located within two different layers (Z planes) across the depth of the sample.

### 4.3 Simultaneous Ca^2+^ imaging of ICC and SMCs located across two tissues layers during colonic contractions

**FIGURE 5:**
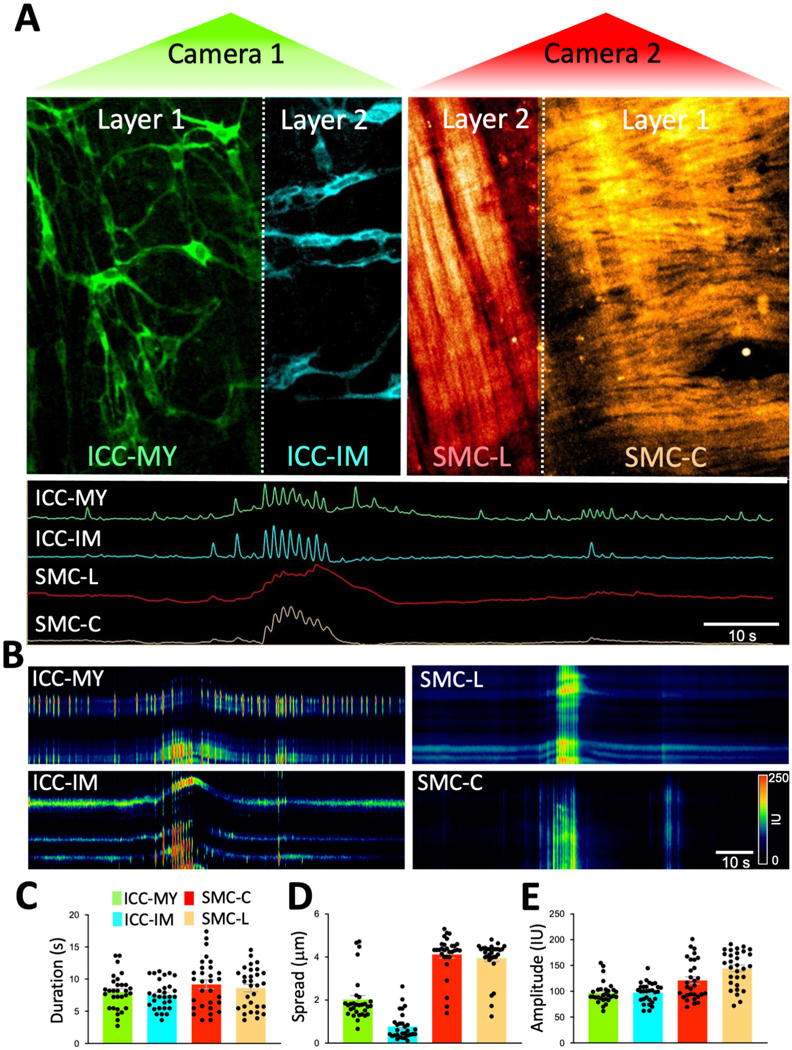
Real-time Ca^2+^ imaging of four cell types organized in two distinct tissue layers simultaneously. **(A)** After applying the piezo bender to the sample to achieve proper imaging of two different Z planes of the colon indicated by Layer 1 and 2 (separated by the dotted white line in each camera) and corresponds to the myenteric region (Layer 1) and longitudinal muscle region (layer 2) within the colon. On one camera (camera 1-green) imaging of GCaMP6f signals were obtained from two types of interstitial cells of Cajal (ICC). ICC in the myenteric region (ICC-MY; layer 1) and intramuscular ICC (ICC-IM; layer 2). These cells are located in two anatomically different Z planes. On the other camera (camera 2-red), RCaMP1.7 signals were collected from smooth muscle cells in longitudinal region (SMC-L; Layer 2) and smooth muscle cells in circular region (SMC-C; Layer 1) these cells are located in two anatomically different Z planes. Note: layer 1 have ICC-MY and circular SMCs whereas layer two have ICC-IM and longitudinal SMCs. During a colonic migrating motor complex (CMMC), Ca^2+^ signals fired from all four different cell types in the two layers (ICC-MY and SMC-C in layer 1and ICC-IM and SMC-L in layer 2). Ca^2+^ transients were plotted on spatiotemporal maps (STMaps) to allow visualization of Ca^2+^ activity in these four types of cells **(B)**. Ca^2+^ event parameters from each cell type were tabulated during a CMCC complex, Ca^2+^ event duration (s) **(C)**, spatial spread (μm) **(D)**, and fluorescence amplitude (Intensity Unit: IU) (E).

Colonic muscles can generate propulsive contractions to move contents in an oral to anal direction. This motility behavior is known as colonic migrating motor complex (CMMC; Koh et al., 2022; Smith et al., 2014). Information on how different cells function and coordinate at different layers within the muscle during CMMC is not known due to limitations in the current imaging approaches. Our method overcomes these limitations by allowing the visualization of Ca^2+^ activity in four types of cells, organized in two layers (Z planes), simultaneously. Imaging of ICC-MY in the myenteric region, ICC-IM within the longitudinal region and imaging of circular and longitudinal SMCs is shown in Figure 5 and Supplementary Video S1. Under basal conditions, ICC-MY fired stochastic, localized Ca^2+^ transients, that occasionally organized into a Ca^2+^ flash across the network (Figures 5A, B). ICC-IM fired stochastic Ca^2+^ transients as discrete events (Figures 5A, B). SMCs in the circular and the longitudinal muscles showed little basal Ca^2+^ activity, but when Ca^2+^ flashes occurred in ICC-MY, Ca^2+^ signals were resolved in SMCs. When CMMC were initiated, Ca^2+^ transients were coordinated in all four cell types as seen in Figures 5A, B and Supplementary Video S1. Ca^2+^ events were tabulated as shown in Figures 5C–E and Table 2. During CMMCs both ICC-MY and ICC-IM respond and fire Ca^2+^ transients that can initiate a Ca^2+^ events in both circular and longitudinal smooth muscles.

In summary, the novel approach described allows real-time, high-resolution imaging across multiple tissue planes simultaneously, via the integration of confocal microscopy and piezo actuators. This method facilitates examination of cell-to-cell coordination in varying tissue depths. The study demonstrates, for the first time, how multiple cell types within the muscle wall of the colon behave and coordinate their functions to produce colonic motor complexes and motility patterns.

## 5 Discussion

This study demonstrates the feasibility of a new approach for imaging multiple tissue layers across the thickness of samples simultaneously, providing a clear visualization and cost effective method to enhance research imaging capabilities as summarized in Supplementary Table S1. We combined confocal microscopy, which is widely used in research laboratories, piezo actuators and fluorescent signals (optogenetic sensors) to achieve high resolution imaging of distinct tissue layers simultaneously. Tissues are typically organized in layers and one major obstacle is the ability to image dynamic fluorescent signals in cells from layers within tissue depths simultaneously. Such information is valuable and answers questions regarding cell-to-cell communication, coordination, and how each type of cell contributes to integrated responses to physiological stimuli.

In this paper, we demonstrated the feasibility of this new approach, by utilizing an inherent feature of confocal microscopy that allows visualization of samples in the z dimension. Combining this with the new line of commercially available piezo actuators, we were able to manipulate the height of the sample to bring two district layers (2 different Z planes) into the focal imaging plane. This configuration, along with the use of fluorescent markers (i.e., GFP) or dynamic signals (i.e., GCaMP to observe Ca^2+^) allows simultaneous monitoring of cells located at two different planes in real-time. Using our method, we show that static and dynamic fluorescent signals can be monitored and resolved in an unprecedented way. Employing optogenetic reporter mice allowed us to image fluorescently tagged ICC and SMCs and dynamic Ca^2+^ signals (GCaMP and RCaMP) in these cells *in situ* located at different planes (layers) in the same FOV.

The ability to monitor Ca^2+^ activity in multiple cell types and tissue layers simultaneously offers a new means of understanding how cell-to-cell interactions drive GI motility. Our findings highlight the critical role of ICC in coordinating Ca^2+^ signaling across the gut wall, influencing SMC behavior and contributing to the generation of complex colonic motility patterns, such as CMMCs. Neurogenic and myogenic components are responsible for CMMCs, but questions remain about which cell types are critically activated during CMMCs, how circular or longitudinal muscle cells respond during CMMCs. Interstitial cells of Cajal (ICCs) are specialized cells in the gastrointestinal tract that serve as pacemakers and mediators of neural inputs to smooth muscle cells. ICCs are integral for the initiation and propagation of these motor patterns (Baker et al., 2021; Sanders et al., 2024; Drumm et al., 2022; Sanders et al., 2023; Koh et al., 2022; Bayguinov et al., 2010; Baker et al., 2025). They generate rhythmic electrical slow waves, which coordinate muscle contractions and help synchronize the activity of smooth muscle cells. The proper functioning of ICCs is crucial for the normal operation of CMMCs, as disruptions in ICC activity can lead to motility disorders, such as slow transit constipation (He et al., 2000; Wang et al., 2008; Lyford et al., 2002). ICC functions rely on Ca^2+^ signaling because they express a dominant Ca^2+^-activated Cl^−^ conductance encoded by *Ano1*. Activation of ICC causes depolarization that is conducted to SMCs via gap junctions and activates Ca^2+^ influx through voltage-dependent Ca^2+^ channels and contraction (Sanders et al., 2024; Drumm et al., 2022; Sanders et al., 2023). A recent report documents the importance of ICC in initiation of CMMCs (Koh et al., 2022). Here we show that ICC-MY in the myenteric region and ICC-IM in longitudinal muscle contribute to initiation of network-wide Ca^2+^ transients that lead to activation of CMMCs. SMCs also respond to the Ca^2+^ transients in ICC-MY by initiation of Ca^2+^ transients and subsequent contraction. The temporal and spatial characterization of these events were identified in the present study using the technique introduced. The imaging approach developed in this study can be applied to other complex tissues, providing a powerful tool for investigating cellular communication and function in various physiological and pathological conditions.

In conclusion, the new high resolution microscopic confocal imaging of dynamic fluorescence signals in deep tissue approach described represents a significant advancement in the study of tissue-level cell-to-cell communication. By enabling high-resolution, real-time imaging of multiple cell types within different tissue layers, this technique offers new opportunities for understanding the complex interactions that underlie GI motility and other physiological processes.

## Supplementary Material

Movie 1 SUPPLEMENTARY VIDEO S1
Ca^2+^ imaging of four cell types in two district tissue layers simultaneously. Ca^2+^ imaging was acquired using colonic segments that express GCaMPf6f in Kit^+^ cells (Ca^2+^ sensor; green) and RCaMP1.7 in SMCs (Ca^2+^ sensor; red). Sample fluorescence emissions were collected on two cameras simultaneously after applying the piezo bender to the sample to achieve proper imaging of 2 planes of the colon (myenteric and circular muscle regions). On one camera imaging of GCaMP6f signals were obtained from two types of ICC (color-coded: ICC-MY green, and ICC-IM cyan). These cells are located in 2 anatomically different Z planes. On the other camera, RCaMP1.7 signals from SMCs were collected from longitudinal smooth muscle (color-coded: SMC-L in red) and circular smooth muscle (color-coded: SMC-C in orange) that are located on 2 Z planes similar to ICC. During a colonic migrating motor complex, Ca^2+^ signals fired from these 4 cell types. Ca^2+^ transients traces were plotted to allow visualization of Ca^2+^ activity in these 4 types of cells simultaneously.

Table S1 SUPPLEMENTARY TABLE S1
Comparison of key elements of conventional confocal 3D imaging vs. 2 layer imaging method for dynamic signals.

The Supplementary Material for this article can be found online at: https://www.frontiersin.org/articles/10.3389/fimag.2025.1538533/full#supplementary-material

## Figures and Tables

**TABLE 1:** Average distances between the four types of ICC in the colon.

ICC location	Distance (μm)	ICC location	Distance (μm)
ICC-SM to ICC-IM	17.4 ± 1.9 μm, *n* = 8	ICC-IM to ICC-MY	14.7 ± 1.2 μm, *n* = 11
ICC-SM to ICC-MY	32.4 ± 4.1 μm, *n* = 8	ICC-IM to ICC-SS	25.2 ± 2.4 μm, *n* = 7
ICC-SM to ICC-SS	42.1 ± 4.1 μm, *n* = 7	ICC-MY to ICC-SS	10.3 ± 0.8 μm, *n* = 7

Interstitial cells of Cajal located in the submucosal region (ICC-SM), located in the intramuscular region (ICC-IM), located in the myenteric plexus region (ICC-MY) and in the outer serosal layer (ICC-SS).

**TABLE 2:** Ca^2+^ events parameters in four types of cells in the colon.

Cell type	Firing duration (s)	Spatial spread (μm)	Signal amplitude (IU)
ICC-MY	8.01 ± 0.49	2.00 ± 1.1	97.00 ± 20.9
ICC-IM	7.52 ± 0.40	0.77 ± 0.6	97.40 ± 10.1
Circular SMCs	9.18 ± 0.69	4.12 ± 0.9	121.3 ± 38.0
Longitudinal SMCs	8.61 ± 0.58	3.90 ± 0.9	144.5 ± 34.7

Interstitial cells of Cajal located in the in the myenteric plexus region (ICC-MY), ICC located in the intramuscular region (ICC-IM), smooth muscle cells (SMCs) located in the circular muscle layer and SMCs located in the longitudinal muscle layer (*n* = 5; 30 events).

**Table:** Key resources table

REAGENT or RESOURCE	SOURCE	IDENTIFIER
Experimental models: Organisms/strains		
GCaMP6f-floxed mice (B6;129S-*Gt(ROSA)26Sor*^*tm95.1(CAG-GCaMP6f)Hze*^/J)	Jackson Laboratories	Strain #:024105 RRID:IMSR_JAX:024105
Kit-Cre mice (c-Kit^+/Cre-ERT2^)	Provided by Dr. Dieter Saur	https://doi.org/10.1113/JP271699
Software and algorithms		
Fiji, version 2.0.0-rc-69/1.52	NIH	https://fiji.sc/
4SM, Subcellular Fluorescence Analysis Software	GitHub	https://doi.org/10.1016/j.isci.2022.104277 ; https://github.com/SharifAmit/CalciumGAN/tree/main
STMapAuto, Ca^2+^ Analysis plugin	GitHub	https://doi.org/10.1016/j.ceca.2020.102260

## Data Availability

The raw data supporting the conclusions of this article will be made available by the authors, without undue reservation.

## Abbreviations:

FOV: Field of view
GI: Gastrointestinal
ICC: Interstitial Cells of Cajal
ICC-IM: Intramuscular interstitial cells of Cajal
ICC-DMP: Interstitial cells of Cajal at the deep muscular plexus region
ICC-MY: Interstitial cells of Cajal at the myenteric plexus region
ICC-SM: Interstitial cells of Cajal at the submucosal region
ICC-SS: Interstitial cells of Cajal at the serosal region
GCaMP: Genetically encoded Ca^2+^ indicator composed of a single GFP
KRB: Krebs Ringer Bicarbonate
ROI: Region of interest
STMap: Spatio-Temporal Map
