# Supplementary material for: Novel imaging approach for simultaneous tracking of cell dynamics in distinct tissue layers reveals cells involved in colonic peristalsis
Source: Front Imaging. Author manuscript; Available in PMC 2026 Jan 27. (PMC12834056; doi:10.3389/fimag.2025.1538533)
Supplement: Table S1 — SUPPLEMENTARY TABLE S1 Comparison of key elements of conventional confocal 3D imaging vs. 2 layer imaging method for dynamic signals. [file NIHMS2119663-supplement-Table_S1.pdf]

	Conventional 3D Imaging	2-Layer Imaging Method
Acquisition mode	Sequential	Real-time
Focal plane	Only one layer imaging in the focal plane at any given time	2 layers in the same focal plane
Time delay	Acquisition time delay between the 2 layers	NO acquisition time delay
Static image acquisition	YES	YES
Dynamic Signal acquisition	YES (with time delay)	YES
Dynamic Signal 2-layers in real time	NO	YES
